# A Comprehensive Physical Impedance Model of Polymer Electrolyte Fuel Cell Cathodes in Oxygen-free Atmosphere

**DOI:** 10.1038/s41598-018-23071-5

**Published:** 2018-03-21

**Authors:** Michael Obermaier, Aliaksandr S. Bandarenka, Cyrill Lohri-Tymozhynsky

**Affiliations:** 1 BMW Group, 80788 Munich, Germany; 2 Energy Conversion and Storage – ECS, Physik-Department, Technische Universität München, James-Franck-Straße 1, 85748 Garching, Germany; 3 Nanosystems Initiative Munich (NIM), Schellingstraße 4, 80799 Munich, Germany

## Abstract

Electrochemical impedance spectroscopy (EIS) is an indispensable tool for non-destructive operando characterization of Polymer Electrolyte Fuel Cells (PEFCs). However, in order to interpret the PEFC’s impedance response and understand the phenomena revealed by EIS, numerous semi-empirical or purely empirical models are used. In this work, a relatively simple model for PEFC cathode catalyst layers in absence of oxygen has been developed, where all the equivalent circuit parameters have an entire physical meaning. It is based on: (i) experimental quantification of the catalyst layer pore radii, (ii) application of De Levie’s analytical formula to calculate the response of a single pore, (iii) approximating the ionomer distribution within every pore, (iv) accounting for the specific adsorption of sulfonate groups and (v) accounting for a small H_2_ crossover through ~15 μm ionomer membranes. The derived model has effectively only 6 independent fitting parameters and each of them has clear physical meaning. It was used to investigate the cathode catalyst layer and the double layer capacitance at the interface between the ionomer/membrane and Pt-electrocatalyst. The model has demonstrated excellent results in fitting and interpretation of the impedance data under different relative humidities. A simple script enabling fitting of impedance data is provided as supporting information.

## Introduction

In the Paris agreement, 195 countries agreed on “holding the increase in global average temperature well below 2 °C above pre-industrial levels”^1^. About 14% of the global emission of CO_2_, one of the dominating greenhouse gases, is due to transportation^2^. Electromobility is thought to be a key development to enable a reduced carbon footprint in the transportation sector. Fuel cell electric vehicles (FCEVs), possessing large driving ranges (>500 km) and short refueling times (<5 min), are one of the promising candidates for a driving without exhausting greenhouse gases like CO_2_. FCEVs use Polymer Electrolyte Fuel Cells (PEFCs) to convert chemical energy of the stored hydrogen into electrical energy, which is used to drive the electric engine. Besides the cost reduction, the main focus of PEFC research is on the increase of fuel cell efficiency. Much of the efficiency losses in PEFCs can be accounted to the cathode catalyst layer (CCL) because of both slow oxygen reduction kinetics^3–6^ and proton conduction losses within the catalyst layer (CL). The latter is especially important at low relative humidity. Both kinetics of the oxygen reduction reaction and proton resistance within the CL can be investigated by means of electrochemical impedance spectroscopy (EIS), which is one of the main techniques in PEFC research. Due to the fast hydrogen oxidation reaction kinetics^7^ and the assumed absence of any reactive gases at the cathode, the impedance spectrum of a PEFC operated with hydrogen and nitrogen can exclusively be described by the ohmic resistance of the cell *R*_ohm_, the ionomer conductivity within the CL *κ*_*CL,i*on_, and the specific double layer capacitance at the I/C-interface c_dl_^8–10^. A real CL has a porous structure consisting of carbon particles and ionomer forming agglomerates, which create pores of different sizes^11^. Moreover, the Pt catalyst itself contributes significantly to the impedance response. Therefore, impedance modeling with an entire physical meaning of all parameters of the model is challenging. However, a clearer understanding of the EIS response of PEFC cathodes is of paramount importance.

Nowadays, there are several approaches to account for the CL’s porous structure as well as for various phenomena at the PEFC’s CCLs. These approaches usually use semi-empirical or totally empirical model elements or methodologies, for instance so-called constant phase elements (CPEs)^12–15^, macro-homogenous models^16–19^ or fractal models^20,21^. However, CPEs have normally no clear physical meaning. While analytical and numerical models^19,22–33^ possess a high degree of complexity, alternative approaches are usually not providing a full physical description of the system or do not show an acceptable fitting within the whole frequency range required for the fuel cell impedance tests.

In this work, a fully physical impedance model for PEFC CCLs in absence of O_2_ has been developed and proposed to use in PEFC impedance analysis. The modeling is based on five pillars, which account for the independent experimental data on the pore properties, de Levie’s analytical formula to calculate the impedance response of a single cylindrical pore, the ionomer distribution in a single pore, the specific adsorption of sulfonate groups (from Nafion polymer) at the surface of the Pt-catalyst and possible side reactions (e.g. caused by hydrogen crossover through the membrane). The developed model has no CPE elements and has demonstrated excellent results in fitting and interpretation of the impedance responses characterizing the interface between the ionomer (membrane thickness ~15 μm) and Pt-electrocatalyst supported on carbon at the cathode side under different humidities.

## Experimental

**Figure 1:**
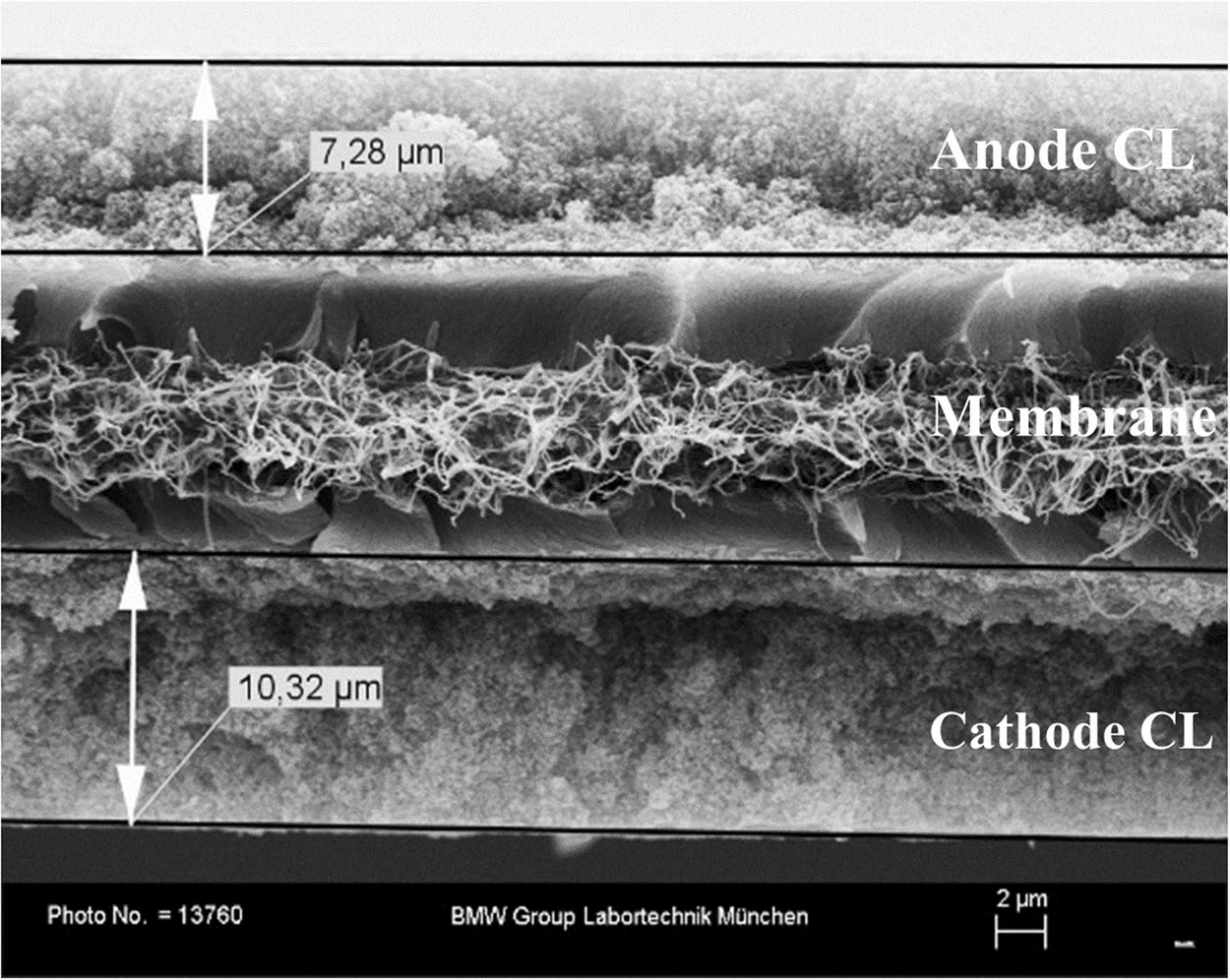
Scanning electron microscopy of an example CCM, showing the thickness ofthe anode catalyst layer, membrane, and the cathode catalyst layer.

A FuelCon Evaluator C-1000-LT test station was used to operate the fuel cells. AC-impedance measurements were conducted using a Zahner electrochemical workstation IM6 together with a potentiostat PP241. A state-of-the-art high-performance membrane electrode assembly (MEA) provided by Johnson Matthey plc with an active area of 43 cm^2^ was placed between two gold-plated flow field plates in cross-flow arrangement. During the measurement using H_2_/N_2_ mode, the anode was fed with H_2_ serving as the analog of the reversible hydrogen electrode (RHE). The cathode was fed with nitrogen. The cathode potential was set at 0.5 V *vs* the anode potential (0 V). The probing AC signal amplitude was set to 20 mV, and a frequency range from 20 kHz to 20 mHz was chosen. The choice of the amplitude value was based on a compromise between a good signal-to-noise ratio and linearity of the system. Using standard Krammers-Krönig tests and Fourier transform analysis it has been found that 20 mV amplitude was the optimal to satisfy both criteria. Ten frequency points were recorded per decade and each measurement point was measured four times and averaged. For each condition, two spectra were recorded in order to ensure quasi-stationarity. The gas and cell temperatures were set to 80 °C. The setup was flushed with nitrogen for about 2 h trying to remove oxygen from the fuel cell and the test station before starting the measurement. In order to investigate the impedance’s dependence on the relative humidity (RH), the bubblers of the anode and cathode side were set to dew point temperatures of T_d_ = 44.8 °C 59.0 °C, 67.9 °C 74.6 °C and 80 °C corresponding to RH = 20%, 40%, 60%, 80% and 100% respectively. In order to keep the partial pressures of the supplied gases constant, the gas outlet pressures were adjusted according to the RH to 1.59 bar, 1.69 bar,1.78 bar, 1.88 bar, and 1.97 bar at RH of 20%, 40%, 60%, 80%, and 100% respectively. This way the reference potential of the anode was maintained. Scanning electron microscopy (Zeiss SUPRA 55VP, 10–20 kV) was used to determine membrane, cathode CL and anode CL thickness of the used CCM (see Fig. 1). Cryo-fracturing of the sample using liquid nitrogen was applied in order to access the sample’s cross-sectional area. The investigated structure was sputtered with gold in order to increase its electronic conductivity. Since the developed model is based on the description of pores being covered with ionomer (pore radius > 10 nm)^34^, mercury porosimetry (QUANTACHROME POREMASTER 60-GT), being able to provide information about the whole relevant pore radius range, was used to quantify pore radius and pore number. Three samples (~6 × 25 cm²) were measured and averaged for further considerations.

Table 1 summarizes further experimental information.

**Table 1:** Parameters describing the MEA used in this work.

Example membrane thickness	~15 µm
Example CCL thickness	~10 µm
Example CCL’s Ionomer/Carbon (I/C) ratio	0.8
Example ionomer density ρ_Ionomer_	2.0 g/cm^3^
Example carbon electrode density ρ_Carbon_	0.4 g/cm^3^

## Results and Discussion

### Model Description

**Figure 2:**
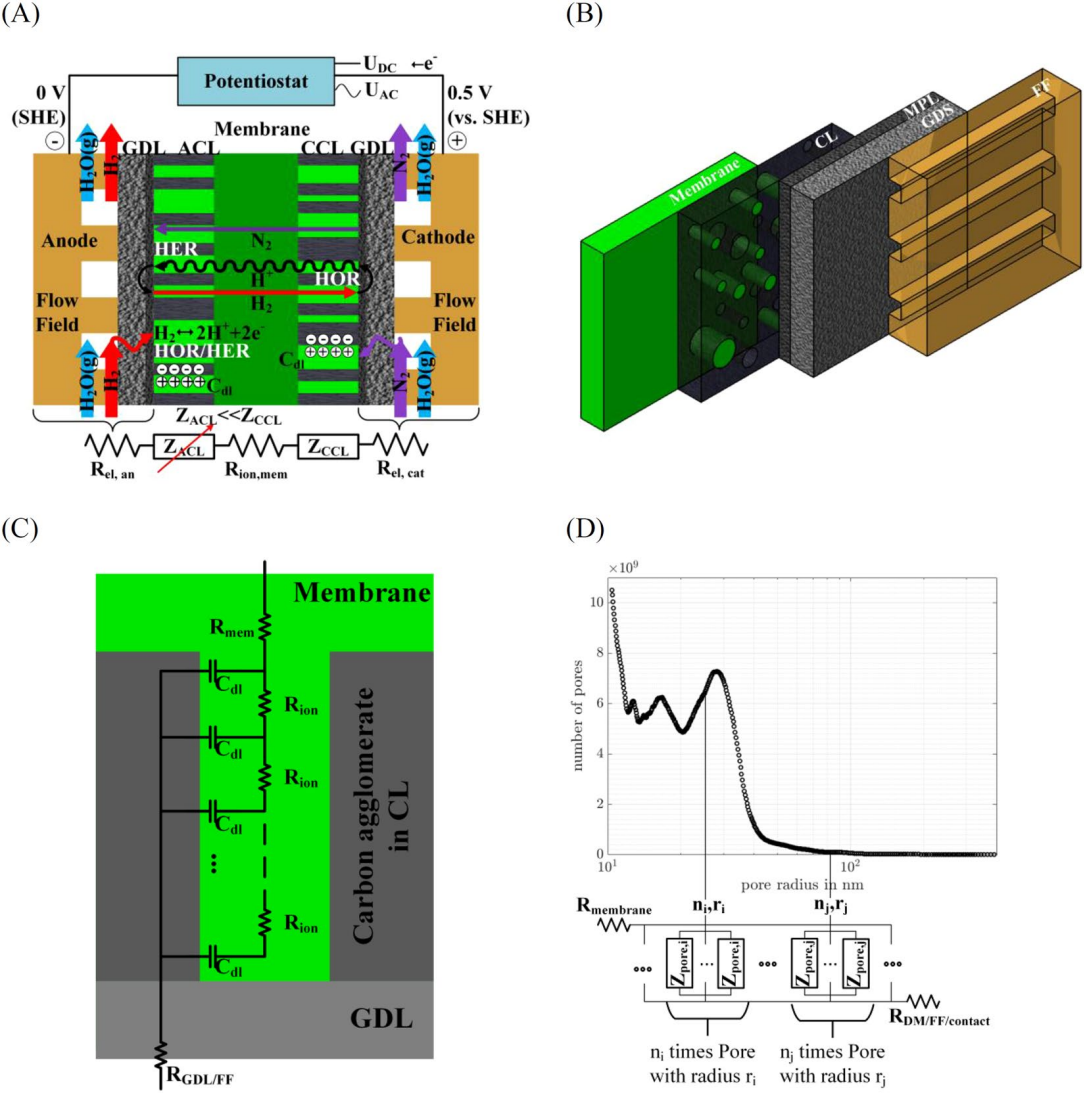
Modeling approach. (**A**) Schematic of processes in a PEFC occurring in the H_2_/N_2_-mode under the AC-probing (e.g. H_2_ oxidation/reduction at anode, H_2_ crossover, double layer charging). EC’s elements for the single components and processes are related to PEFC’s components. (**B**) PEFC’s MEA architecture: from left to right: membrane, CL, GDL (micro-porous layer, MPL, gas diffusion system, GDS), flow field (FF). (**C**) TLM of a single pore completely filled with ionomer consisting of a parallel connection of N double layer capacitances C_dl_ connected via the ionomer resistance R_ion_. (**D**) Quantification of CL’s pore number by means of Hg-porosimetry. The TLM uses the experimental determined pore number and radius to account for impedances of differently sized pores in the CL.

Figure 2A shows the relation between PEFC’s components and the developed model. Electric resistance of flow field (FF) plates and gas diffusion layer (GDL) as well as the ionic resistance of the membrane are modeled by resistive elements R_el_ and R_mem_. Anode’s and cathode’s catalyst layers (ACL and CCL) are modeled by sub-models represented by Z_ACL_ and Z_CCL_, respectively. With the assumption of the absence of any reactive gases, the impedance response of the CCL is given only by the double layer charging of the porous structure. The impedance response of the ACL under the two-electrode configuration is dominated by the hydrogen oxidation reduction. As a result of the fast hydrogen reaction kinetics, the impedance response of the anode can be neglected. The remaining equivalent circuit (EC) is given by a series connection of a resistance, accounting for all ohmic resistances and the sub-model of the CCL.

In order to simplify the CCL model’s description, its explanation is started with five main conceptual constituents.

1. The real porous structure of the fuel cell is taken into account. Therefore, in this model the pore radius distribution experimentally determined by mercury porosimetry was implemented as the input parameter. Without including pore size information, it was not possible to fit the impedance response at lower frequencies.
2. De Levie’s analytical formula is used to calculate the impedance response of a single cylindrical pore.
3. The ionomer distribution within the CL is taken into consideration.
4. The specific adsorption of functional sulfonate groups on Pt electrocatalysts^35^ is taken into account.
5. Side reactions are assumed to occur e.g. of hydrogen being crossed through the membrane or traces of oxygen in the nitrogen gas.

Figure 2B shows the MEA architecture used for the experiments and the modeling. Initially, it is assumed that the catalyst layer can be appropriately modeled as an assembly of cylindrical pores of different radii but equal lengths. Subsequently, Fig. 2A schematically explains the situation, where no background Faradaic reactions are expected. The cathode catalyst layer is approximated by a well-known transmission line model (TLM) where the EC of a single pore consists of the parallel connection of N double layer capacitances C_dl_ connected *via* an ionomer resistance R_ion_. Figure 2C shows the corresponding schematics within a pore, which is completely filled with the ionomer. However, in contrast to the common arbitrary assumption about the pore size distribution, it is proposed that experimental data should be used. Figure 2D shows an example of such experimental assessment based on Hg-porosimetry. The corresponding EC of the CL in the absence of any Faradaic reactions as a parallel connection of the individual pores is quantified by this assessment.

**Figure 3:**
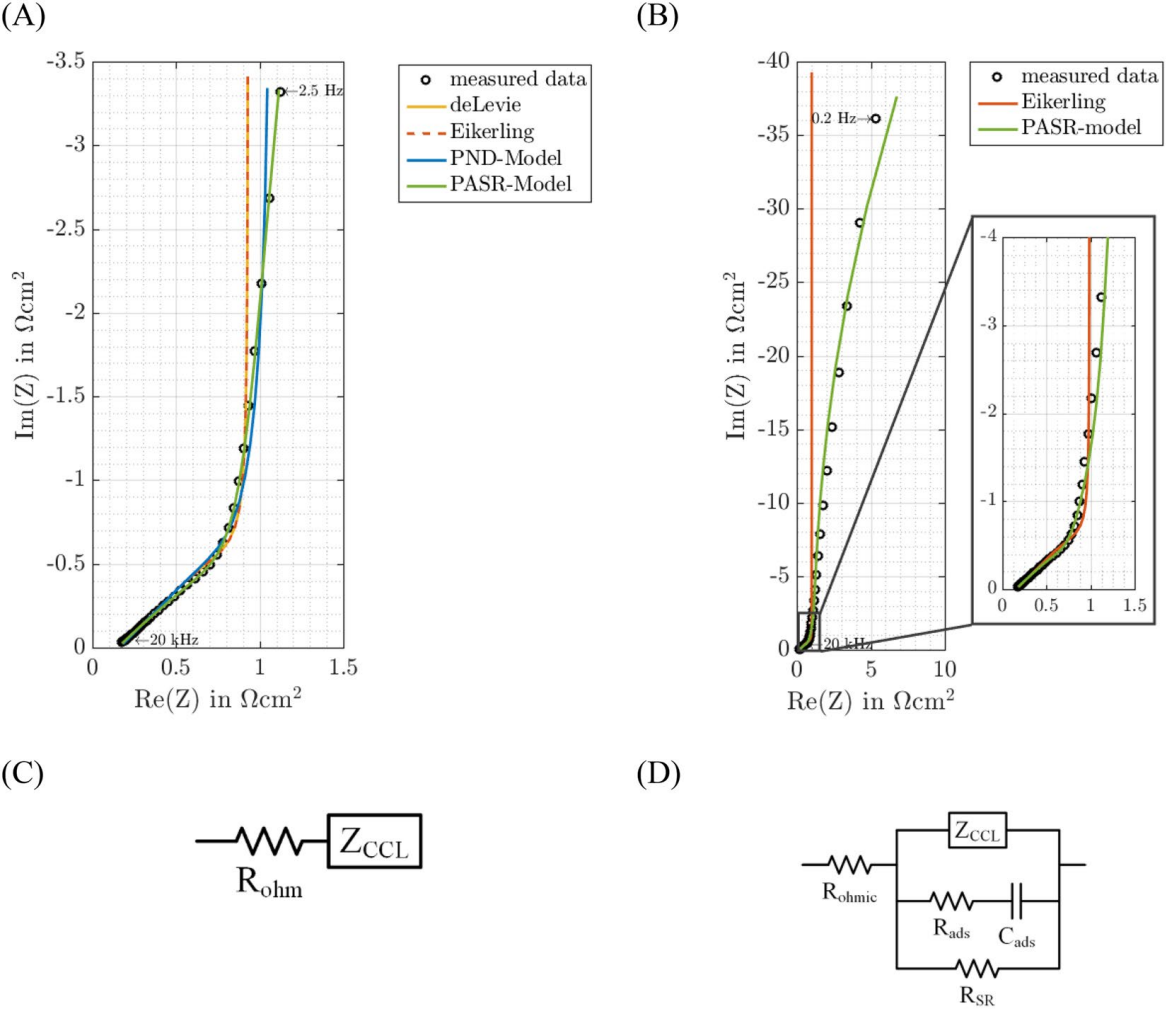
Modeling of the CL. An example of a comparison of modeled and measured impedance data of a 43 cm^2^ fuel cell in the H_2_/N_2_-mode (U_DC_ = 0.5 V, U_AC_ = 20 mV, T = 80 °C, RH = 20%) and the corresponding equivalent circuits. (**A**) Modeling (de Levie, Eikerling, this work) impedance data in the high- and mid-frequency region. (2.5 Hz–20 kHz) using the general equivalent electric circuit given in (**C**), where R_ohmic_ accounts for the uncompensated resistance manifesting itself at the very high frequencies. (**B**) Modeling impedance data in the high-, mid- and low-frequency region (0.1 Hz–20 kHz) considering sulfonate adsorption and side reactions, using the general equivalent electric circuit given in (**D**). Sulfonate adsorption resistance R_ads_ and capacitance C_ads_ as well as resistance of a possible side reaction R_SR_ are included.

It should be noted that the modified transmission line model by means of the experimental pore radius distribution (PTLM) introduced in this work can be considered as consisting of two TLMs accounting for two dispersions in the impedance spectra occurring in the high- and mid- frequency range (see supplementary information). According to the frequency dependent penetration depth λ of the AC-potential perturbation in a pore^36,37^, the spectra in the Nyquist representation should show a straight line in the high frequency range, where real and imaginary parts are equal to each other, thus creating a phase angle of −45° (see an example in Fig. 3A).

The ordinary TLM describes the 45°-region and is given by de Levie’s formula^38^ (see the corresponding fitting in Fig. 3A):

1 $${Z}_{deLevie}=\frac{1}{\sqrt{i{\kappa }_{CL,ion}{{r}_{pore}}^{3}\omega {c}_{dl}}}\,\coth (\sqrt{\frac{2i\omega {c}_{dl}{{l}_{pore}}^{2}}{{\kappa }_{CL,ion}{r}_{pore}}}),$$

where *κ*_*CL,ion*_ is the ionomer conductivity in the CL, *c*_*dl*_ the specific double layer capacitance, *l*_*pore*_ the pore length, *r*_*pore*_ the pore radius, *ω* the angular frequency of the impedance signal and *i* the imaginary unit.

The formula is valid under following general assumptions:

- Electronic resistance of the CL is neglected
- Pore lengths l_pore_ are equal to the thickness of the CCL t_ccl_ multiplied by $\sqrt{\tau \,}$ (tortuosity factor) of the CCL ${{\rm{l}}}_{{\rm{pore}}}={{\rm{t}}}_{{\rm{ccl}}}\cdot \sqrt{\tau }$ (see supplementary information)
- The ionomer/catalyst interface is purely capacitive resulting in an ideal penetration depth
- One-dimensional treatment of pores (due to deep pores)
- No occurrence of Faradaic reactions at the cathode
- Cylindrical pore shape

De Levie’s TLM approach is based on dividing one pore in N slices. Each slice is assumed to have an ideal double layer capacitance at the I/C interface. The ideal capacitances of the single slices are hereby connected in parallel via additional ionomer resistances. Therefore, the capacitances at the electrode side of the pore have a decisive larger ionomer resistance connected in front. When decreasing the frequency, the probing signal penetrates deeper into the pore. Accordingly, this results in an equal increase in imaginary and real part in the Nyquist plot (45° phase shift) followed by a straight 90° line (purely capacitive behavior), as shown in Fig. 3A.

However, taking a further look at the experimental spectrum in Fig. 3A in the mid-frequency range, an obvious non-ideal behavior is depicted as a significant deviation from the 90° degree inclination in the Nyquist representation of the impedance data. This can mainly be attributed to the pore radius distribution in the real sample^36^. Pores of different sizes possess different penetration depth for the applied potential perturbation and, therefore, possess different impedance responses. The parallel connection of these different impedance responses leads to the observed non-ideal behavior. In this work, this is taken into account by using experimental pore radius distribution (Fig. 2C) and using the parallel connection of single pores of different sizes. This significantly improves the fitting, as shown in Fig. 3A.

Considering liquid electrolytes, Robert de Levie assumed pores being completely filled with an electrolyte. This however is not valid for solid electrolytes (ionomer) applied in PEFCs. For PEFCs, a three phase boundary is present in the CCL. According to scanning electron microscopy imaging, the ionomer is assumed to form a homogenous thin film at the pore surface^34^. In addition, ionomer is distributed heterogeneously within the CL forming films of varying thickness^39^. In order to maintain the distribution of single pore’s ionic resistance and to account for the ionomer film thickness distribution within the CL, a pore radius dependent ionomer film thickness (t_ion_ = x*r_pore_) is introduced. According to the ionomer to carbon ratio (I/C) and the density of the carbon structure in the electrode and of the ionomer, the corresponding volume of ionomer in the CCL, V_ionomer_, is calculated and is used together with the determined pore surface area to quantify the ionomer film thickness proportionality factor x (for further details see supplementary information). With respect to the reduction of ionomer cross section area, A_CS_, compared to completely filled pores, the CCL’s ionomer conductivity determined by fitting is reduced according to the definition of conductivity (κ = 1/R*l_eff_/A_CS_). Here R represents the measured impedance and l_eff_ the effective length.

Figure 3C displays the EC accounting for the fuel cell’s ohmic resistance R_ohmic_ and the impedance of the catalyst layer Z_CCL_, which is determined by the use of de Levies analytical formula and the use of the experimental CL’s pore number and ionomer distribution. A detailed derivation of the pore number distribution is given in the supplementary information. In the following, the model being described by this EC will be referred to by pore number distribution model (PND-model). The PND-model shows good fitting performance in the high- and mid-frequency region, outperforming other models used in the literature (see Fig. 3A). However, it must be noted, that the fitting in the low frequency region is rather far from the experimental data (see Fig. 3B). Therefore, further possible processes at the cathode side should be taken into account.

**Figure 4:**
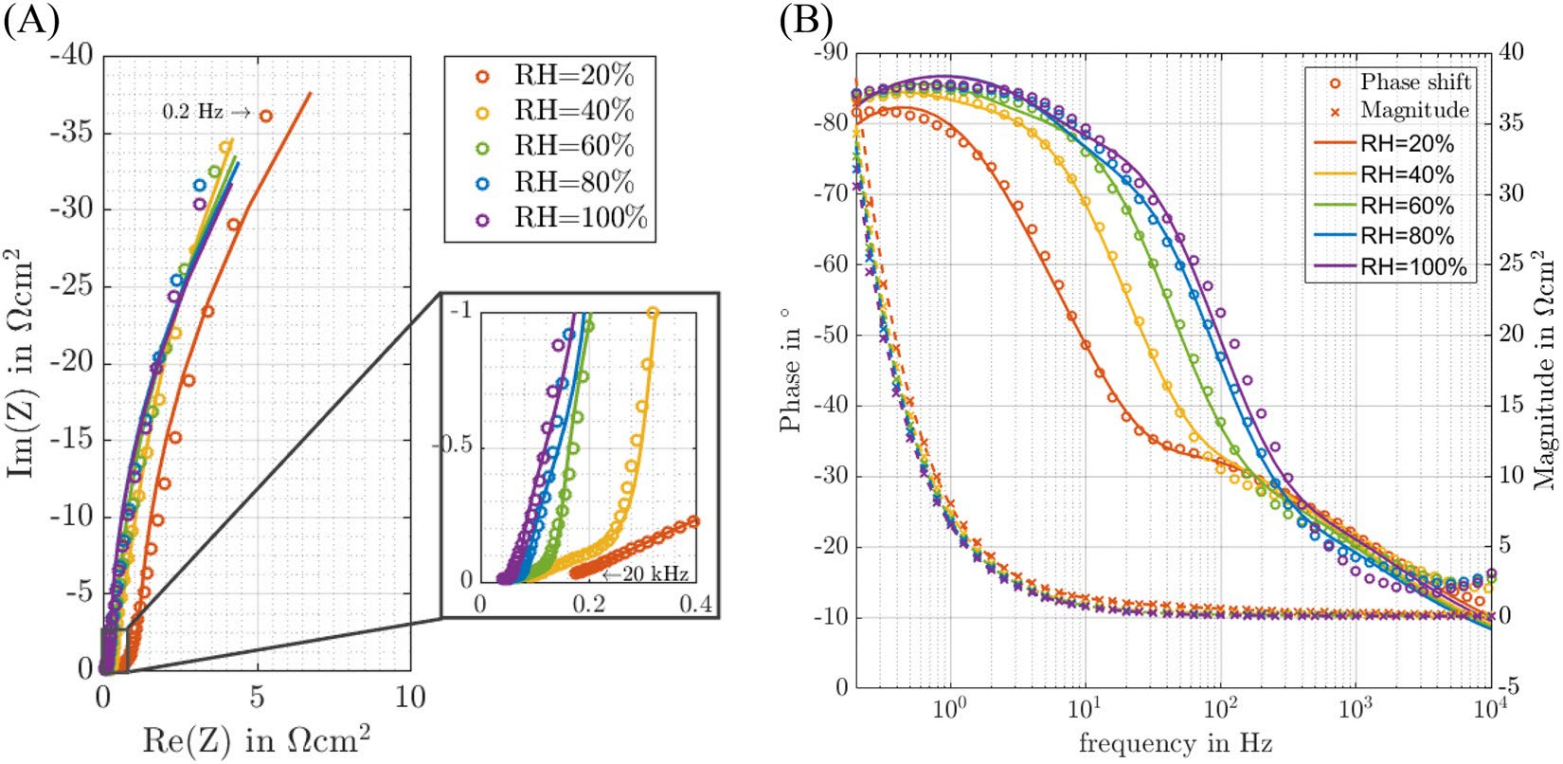
Fitting of the impedance spectra of the CL at different RH using the developed model (equivalent circuit is shown in Fig. 3D). Measured impedance data were obtained from a 43 cm^2^ fuel cell operating in the H_2_/N_2_-mode (U_DC_ = 0.5 V, U_AC_ = 20 mV, T = 80 °C). The pressure was adjusted according to the applied RH. (**A**) Nyquist plot and (**B**) Bode plot show excellent fitting performance up to RH = 60%. For the higher RHs a bit poorer fitting is observed most likely due to ionomer structure changes and capillary condensation.

At the applied working DC-potential close to U = 0.5 V specific adsorption of functional sulfonate groups of the ionomer on Pt-electrocatalysts and specific adsorption of *OH/*O adsorbates coming from water should be taken into account^15,35^. Accounting for the adsorption process, a series connection of adsorption capacitance C_ads_ and adsorption resistance R_ads_ is introduced (see Fig. 3D). Namely this combination of elements represents physical equations describing so-called surface limited and quasi-reversible specific adsorption of anionic adsorbates^33^,^40,41^. Here, R_ads_ = −1/(∂*i*_ads_/∂*E*) and C_ads_ = −q_a_(∂*θ*_ads_/∂*E*); q_a_ is the charge necessary to form an adsorbate layer, *i*_ads_ is the Faradaic current due to e.g. sulfonate group adsorption/desorption, *θ* is the fractional adsorbate surface coverage which “oscillates” around quasi-steady-state values at a given potential under AC-probing. In addition, the occurrence of other (“continuous” in contrast to the surface limited, e.g. hydrogen oxidation) Faradaic side reactions has to be taken into consideration. Therefore, a resistance accounting for possible side reactions, R_SR_ = −1/(∂*i*_SR_/∂*E*), is introduced (*i*_SR_ is the Faradaic current due to side reactions) in parallel, which could be caused by hydrogen cross over through the membrane or traces of oxygen in the nitrogen gas. R_SR_ and series connection of C_ads_ and R_ads_ (classical physical model of reversible surface limited adsorption^42^) are connected in parallel to the catalyst layer model Z_CCL_ as shown in Fig. 3D. The parallel connection is justified by double layer charging, sulfonate adsorption and possible side reactions occurring simultaneously with different time constants. The model being described by the EC accounting for possible side reactions and sulfonate adsorption is in the following referred to by PASR-model (parallel adsorption and side reaction model). Figure 4 shows the improved fitting performance of the PASR-model at the whole frequency range for different relative humidities, as further discussed in the next sections. One should note that effectively, the applied model has only 6 independent fitting parameters (R_mem_, κ_CL,ion_, c_dl,_ R_SR_, C_ads_ and R_ads_) and each of them has clear physical meaning, i.e. no constant phase elements or additional semi-empirical diffusion elements. This is achieved by the use of the experimental pore size distribution and careful accounting for the most probable Faradaic reactions. Table 2 gives an example of values of the fitting parameters obtained by fitting an impedance spectrum. The values of the fitting parameters are in good accordance with literature^43,44^. Please, see the supporting information for a relatively simple MATLAB code used to implement the model.

**Table 2:** Values of fitted parameters determined by fitting a measured impedance spectrum (43 cm^2^-cell, RH = 20%, T = 80 °C, U = 0.5 V, freq = 0.1 Hz–10 kHz) with the PASR-model.

R_mem_ (Ohm*cm^2^)	κ_CL,ion_1/(Ohm*m)	c_dl_ /(F/m^2^)	R_ads_ (Ohm*cm^2^)	C_ads_ (F)	R_SR_ (Ohm*cm^2^)
0.041	4.35	0.49	24.69	0.15	415.94

### Model verification under different experimental conditions

#### Fitting of impedance spectra

Properties of the CCL (i.e. ionic conductivity κ_CL,ion_ and specific double layer capacitance c_dl_ obtained from the fitting) were investigated by fitting the impedance data obtained from EIS-measurements under H_2_/N_2_-atmosphere by means of the PND- and PASR-model. Similar to the model developed by de Levie^38^ and by Eikerling^19^, the developed models perform very good fitting of measured data in the high frequency region (see Fig. 3). Figure 3A shows the superior fitting in the mid-frequency region of the PND-model compared to de Levie’s and Eikerling’s model. With the introduction of C_ads_, R_ads_ and R_SR_ (see Fig. 3D), accounting for sulfonate adsorption and possible side reactions, the PASR-model also performs excellent fitting in all frequency regions (see Fig. 3B).

Figure 4 shows impedance data measured at different relative humidities. The PASR-model demonstrates excellent fitting for humidities up to RH = 60%. At higher RHs the modeled impedance deviates slightly from the measured one, however within few percent of root-mean-square deviation. It is most likely due to the fact that at high RH, ionomer structure can change, resulting in a deviating double layer formation and deviating conductive ionomer channels in the porous structure. Moreover, water formed by means of capillary condensation can create additional double layer surfaces in pores not being covered with ionomer. In addition, water droplets formed in the cell hardware can influence the measured impedance data. In the following sections, a closer look is taken towards the dependencies of the double layer capacitance and the ionic resistance of single pores. In the supplementary information, examples of the fitting related the applicability of the model in the case of structurally different MEAs are also given.

#### Double layer capacitance

**Figure 5:**
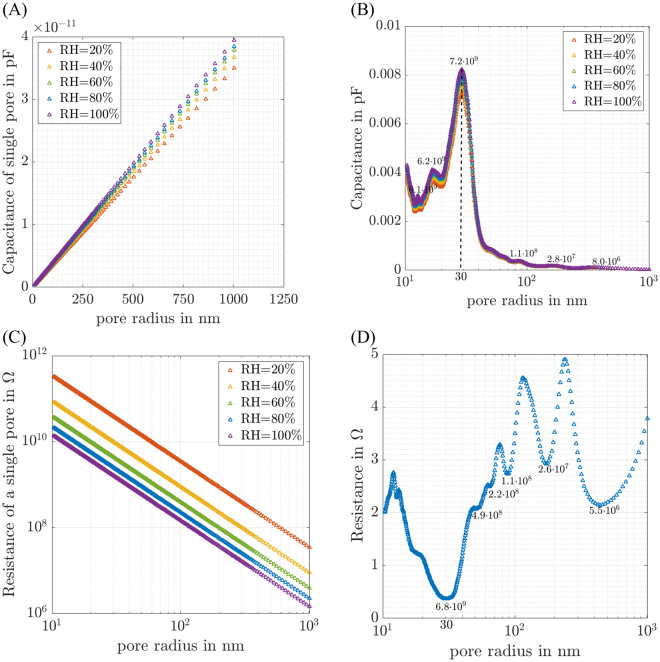
Dependence of CL’s properties on pore radius at different RHs. Dependence on pore radius of (**A**) the double layer capacitance of a single pore. Linear dependence is in accordance with assumption of cylindrical pores. (**B**) The sum of the double layer capacitances of all pores of a given size (number of pores of selected pore radii is displayed in the diagram). Pores in the radius range of 30 nm have the highest impact to the CL’s overall double layer capacitance. (**C**) Ionic resistance of a single pore as a function of the pore radii. Inverse quadratic dependence is in accordance with the assumption of cylindrical pores. (**D**) Ionic resistances related to all pores of a given size (at RH = 80%) (number of pores of selected pore radii is displayed in the diagram).

The single pore’s double layer capacitance C_pore,dl_ at the I/C-interface is given by the product of pore’s surface area A_S_ and CCL’s specific double layer capacitance c_dl_, which was determined by fitting the impedance data. According to the assumption of cylindrical pores of constant length, the pore’s surface area (A_S_ = 2π*r_pore_*l_pore_) and C_pore,dl_ possess linear dependences on the pore radius (see Fig. 5A). With regard to the increasing surface area larger pores possess a higher capacitance. Figure 5B displays the sum of double layer capacitances of all pores of a given size as a function of pore radius. Although the capacitance of the single pores increases with increasing radius, pores in the range of 30 nm have the highest impact towards the overall double layer capacitance of the CCL. This can be explained by means of the CCL’s PRD having its peak pore number in the range of 30 nm (see Fig. 2D). Note however, that the shapes of the curves shown in Figs 2D and 5B are not the same: significant “non-trivial” differences are clearly seen.

Figure 5A,B also shows an increase of specific double layer capacitance with increasing relative humidity. Possible explanations for this effect can be the appearance of additional capacitance from capillary condensation^45^, non-uniform change in the dielectric constant of the double layer^46,47^, or geometric expansion of the ionomer film^48^.

#### Pore resistance

The ionic resistance of a single pore is given by R_pore,ion_ = 1/ κ_CL,ion_*l_pore_/A_CS_, where the cross sectional area of the ionomer is given by A_CS_ = π∙r^2^∙(2x-x^2^). According to this definition, the ionic resistance of a single pore is an inverse quadratic function of pore radius (see Fig. 5C).

The single pore’s ionic resistance increases with decreasing radius. Figure 5D displays the resistance of a parallel connection of all pores of a given size. Pores in the radius range of around 30 nm possess the lowest proton resistance, thus dominating the CCL’s proton resistance. This can once again be explained by means of the CCL’s PRD.

## Conclusions

An all-physical-element impedance model for PEFCs at the presence of hydrogen at the anode and nitrogen at the cathode has been developed. The catalyst layer impedance modeling is based on five pillars, which include application of the de Levie’s formula to describe a single pore’s impedance, experimental quantification of pore number and radii, considering ionomer distribution and taking into account specific adsorption of sulfonate groups at the surface of the Pt-catalyst as well as the occurrence of side reactions such as hydrogen oxidation. It should be emphasized that all parameters used in the model possess physical meaning. Thus, the model is predestinated for PEFC’s CCL analysis, by means of fitting CCL’s characteristic parameters like ionomer conductivity or specific double layer capacitance. Notably, the model has only 6 independent parameters for the fitting. Possible fields of application range from benchmarking and receiving inspection to development-, aging- and failure-analysis. Initial results using one of the state-of-the-art fuel cells at BMW show very promising results. The model performs good fitting in the whole frequency spectrum of impedance data of a PEFC operated in H_2_/N_2_ mode. Considering the assembly of all pores of a given radius (parallel connection), in the particular example used in this work, pores in the radius range of 30 nm have the highest impact towards the CL’s overall double layer capacitance and proton conductivity.

### Data availability

All data including MATLAB code for the fitting are available from the Authors upon request.

## Electronic supplementary material

Supplementary Information

Supporting MATLAB project 1

Supporting MATLAB project 2

Supporting MATLAB project 3

Supporting MATLAB project 4
