# Supplementary material for: A Comprehensive Physical Impedance Model of Polymer Electrolyte Fuel Cell Cathodes in Oxygen-free Atmosphere
Source: Sci Rep. 2018 Mar 21;8:4933. doi: 10.1038/s41598-018-23071-5 (PMC5862870; doi:10.1038/s41598-018-23071-5)
Supplement: Supplementary file 1 — Supplementary Information [file 41598_2018_23071_MOESM1_ESM.docx]

### **Supplementary information to**

***A Comprehensive Physical Impedance Model of Polymer Electrolyte Fuel Cell Cathodes in Oxygen-free Atmosphere***

*Michael Obermaier^1,2^, Aliaksandr S. Bandarenka^2,3,^*, Cyrill Lohri-Tymozhynsky ^1,^**

1- *BMW Group, 80788 Munich, Germany*

2- *Energy Conversion and Storage – ECS, Physik-Department, Technische Universität München, James-Franck-Straße 1, 85748 Garching, Germany*

3- *Nanosystems Initiative Munich (NIM), Schellingstraße 4, 80799 Munich, Germany*

#### **S1: Dispersions in the impedance spectra**

In the high frequency range a first deviation from an ideal capacitive behavior occurs. Different to the ideal spectra the real spectra in Nyquist representation corresponds to a straight line, where real and imaginary part equal each other, thus creating a phase angle of -45° (see fig. 1b). This effect can be described by the frequency dependent penetration depth $\lambda$ of the AC-potential perturbation in a pore ^1,2^.

| 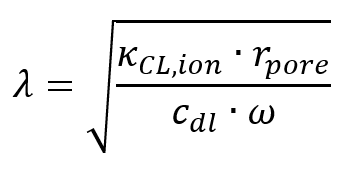 | (S1) |
| --- | --- |

with $\kappa_{CL,ion}$=ionomer conductivity within a pore; ω=angular frequency, $r_{pore}$=pore radius, c_dl_=double layer capacitance at the pore surface. De Levie used a transmission line model (TLM) to account for this in-a-pore-dispersion and derived an analytical formula describing the high frequency dispersion for cylindrical pores ^3^. His approach is a simplification of the concentrated solution theory ^4^. Breaking this complex problem down to a simple formula, de Levi’s approach is widely used in literature ^1,2,5–9^.

In the mid frequency range, a second non-ideal behavior is depicted in a deviation of the ideal capacitive 90° degree inclination in Nyquist representation of the impedance data. Malevich et al. examined this deviation by simulating the impedance spectra of a CL with distributed capacitance and resistance. They concluded that those effects cannot be responsible for the observed deviation ^10^. Song et al. attributed the deviation to a distribution in pore radii (by PRD-dispersion) ^1^. The by-PRD dispersion is based on the dependence of the penetration depth on the pore radius for pores being completely filled with ionomer. Different penetration depths lead to different impedance responses. This means that at a certain frequency the pore radius distribution leads to a distribution in impedance response of single pores and therefore to the observed deviation from the 90° behavior of the overall impedance. They further used this approach to determine the PRD of an electrode from impedance data, which was reconsidered by Musiania et al. ^4^.

#### **S2: Tortuosity factor**

The tortuosity factor $\tau$ of the CL can be calculated with the help of Bruggeman’s expression ^11^:

| 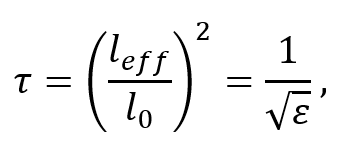 | (S2) |
| --- | --- |

where *l_0_* and *l_eff_* are the edge length of the porous layer and the effective length of the porous channels respectively. The porosity $\varepsilon$ is calculated by means of the hollow volume and the overall volume of the catalyst layer or by means of the CL density and the density of its components.

#### **S3: Pore radius distribution**

With the help of the intruded mercury volume per pressure range (=pore radius range) determined by means of mercury porosimetry ^12,13^, the number of pores in a certain radius interval is calculated. For a volume $\Delta V$being intruded into pores in the pore radius range of [$r-\Delta r$/2, $r+\Delta r$/2] the following relation is valid assuming a cylindrical pore shape and $\Delta r$ being sufficiently small:

| 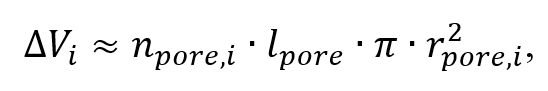 | (S3) |
| --- | --- |

where *n* describes the number of pores in the radius range [$r_{pore,i}-\Delta r_{i}$/2,$r_{pore,i}+\Delta r_{i}$/2] . For sufficiently small radii intervals the intruded volume per pore radius can be written as a function of the linear differential pore volume distribution $D_{V}$:

| 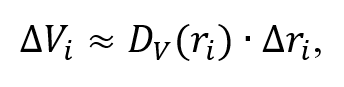 | (S4) |
| --- | --- |

The relative pore number distribution NF is calculated as the ratio of the number of pores of a certain size $n_{i}$:

| 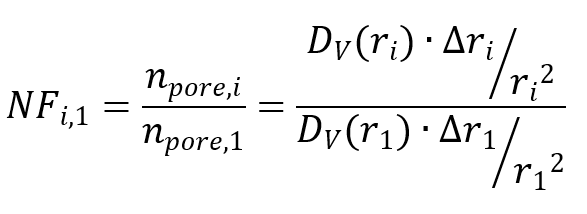 | (S5) |
| --- | --- |
| 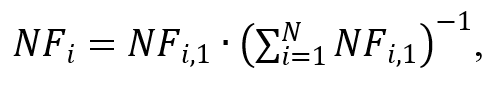 | (S6) |

where N is the number of different measured pore radii in the CL.

The total number of all pores in the CL, *n_tot_*, is given as a function of total intruded volume *V_intr_*:

| 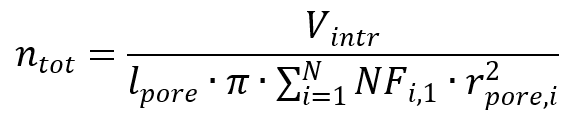 | (S7) |
| --- | --- |

Hereby, a cylindrical pore shape and a unit pore length are assumed as well.

The number of pore *n_pore,i_* of a given radius *r_pore,i_* is finally given by the product of the relative pore population NF and the total number of pore in the CL *n_tot_*:

| 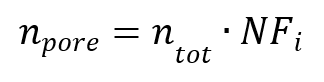 | (S8) |
| --- | --- |

#### **S4: Ionomer distribution**

The thickness *t_ion_=x*r_pore_* of the ionomer film, covering the inner pore surface, is assumed to be a radius dependent value. The value *x* is used as a proportionality constant between ionomer film thickness and pore radius. The proportionality constant *x* is calculated from the CL’s ionomer volume fraction *VF*, which is given as the ratio of total ionomer volume *V_ion_* and total pore volume *V_pores_*:

| 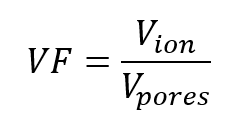 | (S9) |
| --- | --- |

The ionomer volume is calculated as a function of the *I/C* ratio, the effective density of carbon in the electrode *ρ_carb,electr_* ^14^, the ionomer density *ρ_ion_* ^14^ and the overall volume of the electrode *V_electr_:*

| 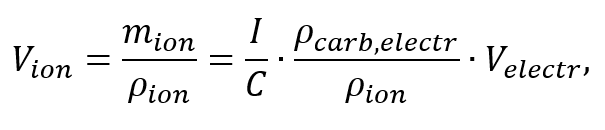 | (S10) |
| --- | --- |

where *m_ion_* describes the ionomer mass in the electrode.

The total pore volume *V_pore_*_s_ is given by the mercury porosimetry measurements.
Since the ionomer is assumed to form a film on the inner pore surface, the ionomer volume
can be described by the pore length *l_pore_,* the pore number distribution and the ionomer film thickness *t_ion_*:

| 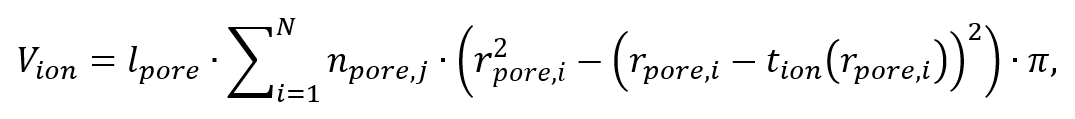 | (S11) |
| --- | --- |

where *N* describes the number of different measured pore radii.

The proportionality constant *x* is therefore given as:

| 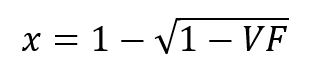 | (S12) |
| --- | --- |

Finally, the radius dependent film thickness is given as:

| 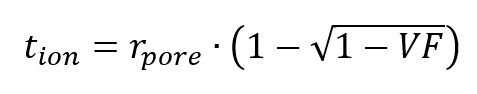 | (S13) |
| --- | --- |

#### **S5: Influence of double layer capacitance on relative humidity**

An increase of the specific double layer capacitance with RH is determined (see Figure 4 in the main text). This can be attributed to several effects: appearance of additional capacitance from capillary condensation, change in the dielectric constant of the double layer or geometric expansion of the ionomer film.

According to Kelvin’s equation ^15^ condensation in small pores can already occur below saturation pressure. The ratio of equilibrium vapor pressure p, at which condensation takes place and the usual saturation vapor pressure *p_0_* is correlated to the radius of cylindrical pores $r_{pore}$:

| 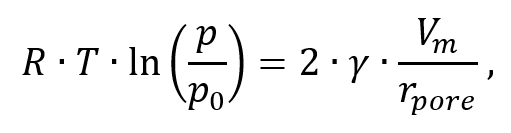 | (S14) |
| --- | --- |

where *R*=ideal gas constant, *T*=temperature in K, *γ*=surface tension and *V_m_*=molar volume of the liquid. With increasing *RH=p/p_0_*, condensation can take place in larger pores. The formed interface between condensed water and carbon causes a double layer capacitance in addition to the double layer capacitance at the I/C-interface.

It was reported that the dielectric constant of ionomer increases with increasing relative humidity ^16,17^. The direct proportionality between dielectric constant and double layer capacitance could also explain the observed dependency.

Ionomer is adsorbing and desorbing water molecules resulting in a corresponding volume change while exposed to humidity. Therefore, I/C interfacial area being responsible for the double layer capacitance is changing accordingly. Morris et al. reported an almost linear in-plane expansion of an ionomer with increasing relative humidity ^18^. The ionomer used by Morris et al. can be considered to be comparable to the one investigated in this work.

#### **S6: Fitted parameters**

| Model | Conditions | κ_CL,ion_/ 1/(Ohm*m) | c_dl_ /  F/m^2^ |
| --- | --- | --- | --- |
| PASR LF | RH=20%, T=80°C, U=0.5V | 0.484 | 0.449 |
| PND LF | RH=20%, T=80°C, U=0.5V | 0.438 | 0.500 |
| Eikerling LF | RH=20%, T=80°C, U=0.5V | 0.363 | 0.494 |
| PASR MF | RH=20%, T=80°C, U=0.5V | 0.426 | 0.403 |
| PND MF | RH=20%, T=80°C, U=0.5V | 0.473 | 0.465 |
| Eikerling MF | RH=20%, T=80°C, U=0.5V | 0.397 | 0.447 |
|  |  |  |  |
| PASR LF | RH=60%, T=80°C, U=0.5V | 4.347 | 0.488 |
| PND LF | RH=60%, T=80°C, U=0.5V | 3.912 | 0.539 |
| Eikerling LF | RH=60%, T=80°C, U=0.5V | 2.826 | 0.536 |
| PASR MF | RH=60%, T=80°C, U=0.5V | 4.265 | 0.497 |
| PND MF | RH=60%, T=80°C, U=0.5V | 4.180 | 0.502 |
| Eikerling MF | RH=60%, T=80°C, U=0.5V | 2.998 | 0.495 |

***Table S1.*** *CL’s parameters determined by fitting with different models. The ionomer conductivity and the double layer capacitance are referred to the ionomer’s cross sectional area and pore’s surface area, respectively. LF and MF indicate a frequency range of 20 kHz to 0.1 Hz and 20kHz to 2.5 Hz respectively.*

#### **S7: Additional experiments for model verification**

In order to prove the applicability of the developed model across a range of electrode constructions, additional experiments were performed on differently structured MEAs. We have carried out experiments on structurally different MEAs of two different suppliers used at BMW. In both cases mercury porosimetry measurements were used to quantify the pore number distribution in the model. For each set of MEAs the model shows good fitting quality of the measured impedance data. As described in the main work, the pore radius and number distribution of the catalyst layer is determined by mercury porosimetry measurement and is given as an input parameter to the developed model in order to fit experimentally measured impedance data of the corresponding MEA. The applicability of the developed model to differently structured MEAs is proven by the good fitting quality observed for both types of MEA. Typical examples are given in Figure 3 of the manuscript and Figure S1.

| (A) | (B) |
| --- | --- |
| 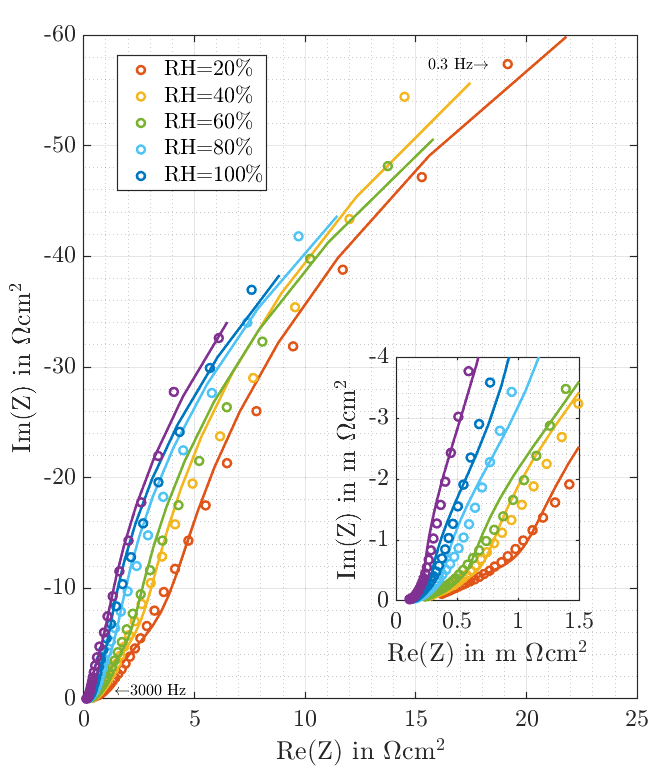 | 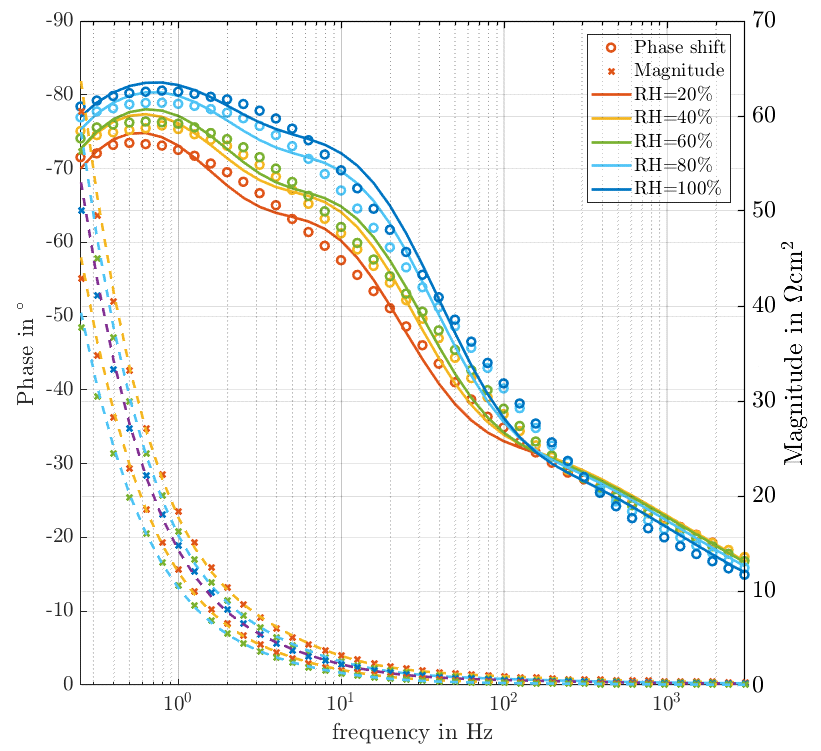 |
| **Figure S1**. Fitting of the impedance spectra of a differently structured CL at different RH using the developed model (equivalent circuit is shown in Figure 2D). Measured impedance data were obtained from a 46 cm^2^ fuel cell operating in the H_2_/N_2_-mode (U_DC_=0.5 V, U_AC_=20 mV, T=80°C). The pressure was adjusted according to the applied RH. (A) Nyquist plot and (B) Bode plot show good fitting quality. | |
|  | |

**References**

(1) Song, H.-K.; Sung, J.-H.; Jung, Y.-H.; Lee, K.-H.; Le Dao, H.; Kim, M.-H.; Kim, H.-N. Electrochemical Porosimetry. *J. Electrochem. Soc.* **2004**, *151*, E102.

(2) Song, H.-K.; Hwang, H.-Y.; Lee, K.-H.; Le Dao, H. The effect of pore size distribution on the frequency dispersion of porous electrodes. *Electrochimica Acta* **2000**, *45*, 2241–2257.

(3) Levie, R. de. Electrochemical response of porous and rough electrodes. *Advances in electrochemistry and electrochemical engineering* **1967**, 329–397.

(4) Musiani, M.; Orazem, M.; Tribollet, B.; Vivier, V. Impedance of blocking electrodes having parallel cylindrical pores with distributed radii. *Electrochimica Acta* **2011**, *56*, 8014–8022.

(5) Barcia, O. E.; D'Elia, E.; Frateur, I.; Mattos, O. R.; Pébère, N.; Tribollet, B. Application of the impedance model of de Levie for the characterization of porous electrodes. *Electrochimica Acta* **2002**, *47*, 2109–2116.

(6) Candy, J.-P.; Fouilloux, P.; Keddam, M.; Takenouti, H. The characterization of porous electrodes by impedance measurements. *Electrochimica Acta* **1981**, *26*, 1029–1034.

(7) Candy, J.-P.; Fouilloux, P.; Keddam, M.; Takenouti, H. The pore texture of raney-nickel determined by impedance measurements. *Electrochimica Acta* **1982**, *27*, 1585–1593.

(8) Cachet, C.; Wiart, R. The pore texture of zinc electrodes characterized by impedance measurements. *Electrochimica Acta* **1984**, *29*, 145–149.

(9) Cachet, C.; Pauli, C. P. de; Wiart, R. The pore texture of zinc electrodes corroded in acidic electrolytes. *Electrochimica Acta* **1985**, *30*, 719–723.

(10) Malevich, D.; Jayasankar, B. R.; Halliop, E.; Pharoah, J. G.; Peppley, B. A.; Karan, K. On the Determination of PEM Fuel Cell Catalyst Layer Resistance from Impedance Measurement in H2/N2 Cells. *Journal of the Electrochemical Society* **2012**, *159*, F888-F895.

(11) Tjaden, B.; Cooper, S. J.; Brett, D. J. L.; Kramer, D.; Shearing, P. R. On the origin and application of the Bruggeman correlation for analysing transport phenomena in electrochemical systems. *Current Opinion in Chemical Engineering* **2016**, *12*, 44–51.

(12) Deutsches Institut für Normierung e.V. *Porengrößenanalyse - Darstellung von Porengrößenverteilungen*, 2009 (66139) (accessed August 23, 2017).

(13) Lowell, S. *Characterization of porous solids and powders: Surface area, pore size and density*, 1. reprint with some corr; Particle technology series 16; Springer: Dordrecht, 2010.

(14) Liu, Y.; Ji, C.; Gu, W.; Jorne, J.; Gasteiger, H. A. Effects of Catalyst Carbon Support on Proton Conduction and Cathode Performance in PEM Fuel Cells. *J. Electrochem. Soc.* **2011**, *158*, B614.

(15) Gregg, S. J.; Sing, K. S. W.; Salzberg, H. W. Adsorption Surface Area and Porosity. *J. Electrochem. Soc.* **1967**, *114*, 279.

(16) Paddison, S. J.; Reagor, D. W.; Zawodzinski Jr, T. A. High frequency dielectric studies of hydrated Nafion®. *Journal of Electroanalytical Chemistry* **1998**, *459*, 91–97.

(17) Paul, R.; Paddison, S. J. A statistical mechanical model for the calculation of the permittivity of water in hydrated polymer electrolyte membrane pores. *The Journal of Chemical Physics* **2001**, *115*, 7762–7771.

(18) Morris, D. R.; Sun, X. Water-sorption and transport properties of Nafion 117 H. *J. Appl. Polym. Sci.* **1993**, *50*, 1445–1452.
